# Circulating *TP53* mutations are associated with early tumor progression and poor survival in pancreatic cancer patients treated with FOLFIRINOX

**DOI:** 10.1177/17588359211033704

**Published:** 2021-08-18

**Authors:** Fleur van der Sijde, Zakia Azmani, Marc G. Besselink, Bert A. Bonsing, Jan Willem B. de Groot, Bas Groot Koerkamp, Brigitte C. M. Haberkorn, Marjolein Y. V. Homs, Wilfred F. J. van IJcken, Quisette P. Janssen, Martijn P. Lolkema, Saskia A. C. Luelmo, Leonie J. M. Mekenkamp, Dana A. M. Mustafa, Ron H. N. van Schaik, Johanna W. Wilmink, Eveline E. Vietsch, Casper H. J. van Eijck

**Affiliations:** Department of Surgery, Erasmus MC, University Medical Center, Rotterdam, The Netherlands; Center for Biomics, Erasmus MC, University Medical Center, Rotterdam, The Netherlands; Department of Surgery, Cancer Center Amsterdam, Amsterdam UMC, University of Amsterdam, Amsterdam, Noord-Holland, The Netherlands; Department of Surgery, Leiden University Medical Center, Leiden, Zuid-Holland, The Netherlands; Isala Oncology Center, Isala Hospital, Zwolle, Overijssel, The Netherlands; Department of Surgery, Erasmus MC, University Medical Center, Rotterdam, The Netherlands; Department of Medical Oncology, Maasstad Hospital, Rotterdam, The Netherlands; Department of Medical Oncology, Erasmus MC, University Medical Center, Rotterdam, The Netherlands; Center for Biomics, Erasmus MC, University Medical Center, Rotterdam, The Netherlands; Department of Surgery, Erasmus MC, University Medical Center, Rotterdam, The Netherlands; Department of Medical Oncology, Erasmus MC, University Medical Center, Rotterdam, The Netherlands; Department of Medical Oncology, Leiden University Medical Center, Leiden, Zuid-Holland, The Netherlands; Department of Medical Oncology, Medisch Spectrum Twente, Enschede, Overijssel, The Netherlands; Department of Pathology, Tumor Immuno-Pathology Laboratory, Erasmus MC, University Medical Center, Rotterdam, The Netherlands; Department of Clinical Chemistry, Erasmus MC, University Medical Center, Rotterdam, Zuid-Holland, The Netherlands; Department of Medical Oncology, Cancer Center Amsterdam, Amsterdam UMC, University of Amsterdam, Amsterdam, The Netherlands; Department of Surgery, Erasmus MC, University Medical Center, Rotterdam, The Netherlands; Department of Surgery, Erasmus University Medical Center, P.O. box 2040, Rotterdam, 3000 CA, The Netherlands

**Keywords:** circulating tumor DNA, FOLFIRINOX, pancreatic cancer, predictive biomarker, TP53

## Abstract

**Background:**

Biomarkers predicting treatment response may be used to stratify pancreatic ductal adenocarcinoma (PDAC) patients for therapy. The aim of this study was to identify circulating tumor DNA (ctDNA) mutations that associate with tumor progression during FOLFIRINOX chemotherapy, and overall survival (OS).

**Methods:**

Circulating cell-free DNA was analyzed with a 57 gene next-generation sequencing panel using plasma samples of 48 PDAC patients of all disease stages. Patients received FOLFIRINOX as initial treatment. Chemotherapy response was determined on CT scans as disease control (*n* = 30) or progressive disease (*n* = 18) within eight cycles of FOLFIRINOX, based on RECIST 1.1 criteria.

**Results:**

Detection of a *TP53* ctDNA mutation before start of FOLFIRINOX [odds ratio (OR) 10.51, 95% confidence interval (CI) 1.40–79.14] and the presence of a homozygous *TP53* Pro72Arg germline variant (OR 6.98, 95% CI 1.31–37.30) were predictors of early tumor progression during FOLFIRINOX in multivariable analysis. Five patients presented with the combination of a *TP53* ctDNA mutation before start of FOLFIRINOX and the homozygous Pro72Arg variant. All five patients showed progression during FOLFIRINOX. The combination of the *TP53* mutation and *TP53* germline variant was associated with shorter survival (median OS 4.4 months, 95% CI 2.6–6.2 months) compared with patients without any *TP53* alterations (median OS 13.0 months, 95% CI 8.6–17.4 months).

**Conclusion:**

The combination of a *TP53* ctDNA mutation before start of FOLFIRINOX and a homozygous *TP53* Pro72Arg variant is a promising biomarker, associated with early tumor progression during FOLFIRINOX and poor OS. The results of this exploratory study need to be validated in an independent cohort.

## Introduction

Although some advances have been made in the treatment of pancreatic ductal adenocarcinoma (PDAC), the prognosis of patients remains very poor.^1,2^ The standard first-line treatment for locally advanced pancreatic cancer (LAPC) and metastatic PDAC is FOLFIRINOX chemotherapy, a combination of fluorouracil, leucovorin, irinotecan, and oxaliplatin. With this treatment regimen, improved overall survival (OS) was observed in both LAPC (24.2 months *versus* 6–13 months)^3^ and metastatic PDAC (11.1 months *versus* 6.8 months)^4^ compared with gemcitabine chemotherapy. FOLFIRINOX is also effective in PDAC patients with stage I–II resectable or borderline resectable disease in the adjuvant setting,^5^ and several clinical trials are investigating the benefit of neoadjuvant FOLFIRINOX followed by surgical resection.^6^

Despite increased survival in patient groups treated with FOLFIRINOX, only a minority of patients will show complete or partial response of the tumor,^7,8^ while approximately 20–30% already develop progressive disease during FOLFIRINOX.^4,9^ Unfortunately, 60–70% of patients experiences severe, grade ⩾4 toxicity from FOLFIRINOX.^3,4,8^ Biomarkers could stratify patients for available therapies. Especially biomarkers that can be measured easily in the circulation, as opposed to tumor tissue, would be ideal. Such a predictive biomarker could prevent non-responding patients from FOLFIRINOX-induced toxicity and select these patients for other treatments.

Circulating cell-free DNA (ccfDNA), including circulating tumor DNA (ctDNA), are short fragments of DNA released into the bloodstream after apoptosis and necrosis of (tumor) cells. CtDNA can be detected in blood serum or plasma, and the presence of tumor mutations in ctDNA is a poor prognostic factor in PDAC patients.^10–12^ Moreover, increasing ctDNA levels over time and the detection of new mutations during chemotherapy are associated with progression of disease.^13–15^ However, most studies focus on *KRAS* mutations only,^13,15^ while several other known cancer-associated gene mutations may indicate PDAC progression and treatment response as well.

The aim of this pilot study was to investigate the value of ctDNA mutations in PDAC patients, detected before the start of treatment or after only one cycle of chemotherapy, to predict early tumor progression during FOLFIRINOX and their association with OS.

## Materials and methods

This article was written according to the reporting recommendations for tumor marker prognostic studies (REMARK) guidelines.^16^

### Patient selection

All patients were selected from two multicenter, prospective trials in the Netherlands. Patients with resectable or borderline resectable PDAC participated in the randomized clinical trial PREOPANC-2 (Dutch trial register NL7094) comparing neoadjuvant FOLFIRINOX with neoadjuvant gemcitabine-based chemoradiotherapy, followed by surgical resection of the primary tumor if applicable. Patients with locally advanced or metastatic PDAC were selected from the prospective cohort study iKnowIT (Dutch trial register NL7522) focusing on the predictive value of circulating biomarkers. Both trials were approved by the ethics committees of all participating hospitals with patients included in this article: Erasmus Medical Center Rotterdam (MEC-2018-087 and MEC-2018-004), Amsterdam UMC, location Academic Medical Center (2018_196 and 2018_138), Leiden University Medical Center (L18.070 and L18.053), Isala hospital Zwolle (180606), and Medisch Spectrum Twente Enschede (H18-081). All patients provided written informed consent and both studies were conducted in accordance with the declaration of Helsinki.

Due to the explorative character of this study, no formal sample size calculation was performed. The authors estimated a sample size of 48 to be achievable and sufficient to draw conclusions from this pilot study. Patient samples were not selected consecutively, but based on the availability of plasma samples and treatment response outcome, in order to have a sufficient number of patients in both investigational groups.

After histological confirmation of the primary tumor and/or metastases, patients from all PDAC disease stages received initial treatment with FOLFIRINOX between February 2018 and September 2019. Patients with resectable, borderline resectable, or locally advanced disease received a maximum of eight cycles of FOLFIRINOX, whereas patients with metastatic disease received a maximum of 12 cycles. Exclusion criteria for patient selection were: age under 18 years, co-treatment with other chemotherapeutics, and previous treatment with FOLFIRINOX. After each fourth cycle of chemotherapy, a computed tomography (CT) scan was performed to evaluate response to treatment according to the Response Evaluation Criteria in Solid Tumours (RECIST) 1.1 criteria,^17^ as part of standard clinical practice. In case of progressive disease, FOLFIRINOX was discontinued. Disease control was defined as stable disease, partial or complete response, and these patients would continue FOLFIRINOX as planned. Patient characteristics, such as age, sex, stage of disease, laboratory results, CT scan evaluations, and follow-up data were retrieved from medical records by a medical doctor. Follow-up ended upon the death of the patient.

### Sample collection

Peripheral venous blood samples were collected before the start of FOLFIRINOX and 2 weeks after the first cycle of FOLFIRINOX. Blood was collected in 10 ml EDTA tubes (Becton Dickinson, Franklin Lakes, NJ, USA) in the Erasmus Medical Center, or 10 ml CellSave tubes (Menarini Silicon Biosystems, Castel Maggiore, Italy) in other centers. CellSave tubes preserve circulating tumor cells and ctDNA up to 96 h at room temperature. CellSave tubes were transferred to the Erasmus Medical Center for processing.

### DNA isolation

Plasma was isolated within 4 h of collection for EDTA tubes or within 72 h for CellSave tubes. For plasma separation, tubes were centrifuged at 1000 *g* for 10 min and again at 1700 *g* for 10 min after transfer into new tubes. Plasma was stored at −80°C until further use.

ccfDNA was isolated from 3 ml of plasma using the QIAamp Circulating Nucleic Acid Kit (Qiagen, Hilden, Germany), according to the manufacturer’s instructions. DNA was eluted in 30 µl buffer AVE (RNase-free water with 0.04% sodium azide) and the eluate was re-applied twice to optimize the DNA yield.

### Next-generation sequencing

ccfDNA concentrations were measured with RT-qPCR quantification using Alu115 primer pairs (Swift Biosciences, Ann Arbor, MI, USA).^18^ The ccfDNA concentrations were derived from the Alu115 RT-qPCR results, representing the total quantity of usable ccfDNA, but excluding short fragments as a result of DNA degradation.

ccfDNA was sequenced using the Accel-Amplicon 57G Plus Pan-Cancer Profiling Panel (Swift Biosciences, Ann Arbor, MI, USA) which covers 286 amplicons of 57 genes. A full gene list is provided in Supplemental Table S1. DNA libraries were prepared using 3–10 ng DNA input, depending on the maximum ccfDNA concentration available. DNA libraries were prepared by multiplex PCR, amplified for 25 cycles in total, followed by the ligation of Illumina adaptors with sample-specific indices. These libraries were pooled and sequenced on an Illumina MiSeq sequencer (Illumina, San Diego, CA, USA), paired-end, with reads of 150 base pairs in length. Fastq files were uploaded in the online Genialis software platform (Genialis, Houston, TX, USA) to trim adaptors of the read and to align the reads to the Human Genome hg19 and to perform LoFreq variant-calling. As a control, four DNA samples were included to assess the consistency of mutation detection: Quantitative Multiplex DNA Reference Standard HD701 (Horizon Discovery, Waterbeach, UK), ccfDNA isolated from ctDNA Ref v2 AF2% plasma (Seraseq, Gaithersburg, MD, USA), ccfDNA isolated from ctDNA Ref v2 WT plasma (Seraseq), and ccfDNA isolated from plasma of a patient diagnosed with lung carcinoma and previously sequenced with IonTorrent sequencing method (ThermoFisher Scientific, Waltham, MA, USA). For mutation calling, several criteria were used: only non-synonymous mutations with variant allele frequency >1%, quality score >200, ⩾10 reads in total and ⩾5 reads per strand, and Fisher strand bias <100 were called mutations. Mutations with allele frequencies ~50% (heterozygous) and ~100% (homozygous) were considered germline mutations, not ctDNA mutations. All base changes and accompanying amino acid changes were annotated on the forward strand.

### Statistical analyses

Continuous data with a non-normal distribution were compared with either Mann–Whitney *U* tests, or with Wilcoxon Signed Rank tests for paired data. Categorical data, such as detection rates of mutations, were compared using Fisher’s exact or Pearson’s Chi-squared tests where appropriate.

Univariable and multivariable binary logistic regression was performed to analyze the predictive value of ccfDNA concentrations and ctDNA mutations for tumor progression during FOLFIRINOX chemotherapy, adjusted for known predictive patient characteristics: stage of disease, and CA19-9 levels. Multicollinearity between variables was checked using the variance inflation factor (VIF). Factors with *p* < 0.10 and VIF < 3 were selected for multivariable analysis.

OS was calculated as the time between the start of FOLFIRINOX and death. All patients included in this cohort died of cancer progression. Differences in median OS were derived from Kaplan–Meier curves whereby groups were compared using log-rank tests. The prognostic value ctDNA mutations was also tested with univariable and multivariable Cox regression analyses, including known prognostic factors: age, stage of disease, and CA19-9 levels. Multicollinearity between variables was checked using the variance inflation factor (VIF). Factors with *p* < 0.10 and VIF < 3 were selected for multivariable analysis.

Only two-sided tests were used and *p-*values < 0.05 were considered statistically significant. Data were analyzed using SPSS Statistics for Windows (version 25.0; IBM, Armonk, NY, USA).

## Results

### Patient characteristics

In total, ccfDNA isolated from plasma from 48 patients was sequenced both before and after the first cycle of FOLFIRINOX, resulting in 96 samples. A total of 18 patients had resectable or borderline resectable disease, 16 LAPC, and 14 metastatic disease. Of these patients, 18 (37.5%) showed progressive disease during FOLFIRINOX, as presented in Table 1. The progressive disease patient group consisted of six patients with resectable disease, six patients with LAPC, and six patients with metastatic disease. The majority of patients had no detectable ctDNA mutations before (31/48 = 64.6%) or after one cycle of FOLFIRINOX (38/48 = 79.2%).

**Table 1.** Patient characteristics.

	All patients, *n* = 48 (%)
Age (years), mean (range)	64 (41–78)
Sex	
Male	28 (58.3)
Female	20 (41.7)
Stage of disease	
Resectable	18 (37.5)
Locally advanced	16 (33.3)
Metastatic	14 (29.2)
Response to FOLFIRINOX^a^	
Stable disease	24 (50.0)
Partial response	6 (12.5)
Complete response	0 (0)
Progressive disease	18 (37.5)
Response to FOLFIRINOX, dichotomized^a^	
Disease control	30 (62.5)
Progressive disease	18 (37.5)
Time point of CT evaluation progressive disease^a^ (*n* = 18)	
After cycle 1	1 (5.6)
After cycle 2	1 (5.6)
After cycle 3	2 (11.1)
After cycle 4	10 (55.6)
After cycle 8	4 (22.2)
Number of cycles of FOLFIRINOX received, mean (range)	7 (1–12)
Baseline CA19-9 (kU/L), median (IQR)	369 (66–2015)
DNA concentration before the start of FOLFIRINOX (ng/ml plasma), median (IQR)	5.98 (3.59–13.67)
DNA concentration after one cycle of FOLFIRINOX (ng/ml plasma), median (IQR)	11.52 (6.42–18.31)
Number of tumor mutations detected before the start of FOLFIRINOX	
0	31 (64.6)
1	9 (18.8)
2	4 (8.3)
3	4 (8.3)
Number of tumor mutations detected after one cycle of FOLFIRINOX	
0	38 (79.2)
1	7 (14.6)
2	2 (4.2)
3	1 (2.1)

a According to the RECIST 1.1 criteria.

CA19-9, carbohydrate antigen 19-9; IQR, interquartile range.

### CtDNA detection

In disease control patients ccfDNA concentrations increased from 6.32 ng/ml plasma [interquartile range (IQR) 4.15–14.73 ng/ml plasma] before start of FOLFIRINOX to 14.25 ng/ml plasma (IQR 8.57–21.58 ng/ml plasma, *p* = 0.028) after one cycle of FOLFIRINOX. In progressive disease patients concentrations increased from 5.54 ng/ml plasma (IQR 1.95–8.96 ng/ml plasma) before start of FOLFIRINOX to 7.52 ng/ml plasma (IQR 3.13–16.72 ng/ml plasma, *p* = 0.007) after one cycle of FOLFIRINOX, as presented in Supplemental Figure S1. There was no statistically significant difference in ccfDNA concentration before chemotherapy between disease control and progressive disease patients (*p* = 0.074). After one cycle of chemotherapy, the median ccfDNA concentration was significantly lower in patients with progressive disease (*p* = 0.018) (Supplemental Figure S1).

In 27 out of 96 (28.1%) of the sequenced plasma samples, at least one ctDNA mutation was detected, corresponding to samples from 21 out of 48 patients. Supplemental Figure S2 shows an overview of each ctDNA mutation detected per plasma sample in 21 out of 48 patients, including their variant allele frequency (VAF).

ctDNA mutation detection rates did not differ between samples collected before start of FOLFIRINOX (35.4%) and samples collected after one cycle of FOLFIRINOX (20.8%, *p* = 0.112). There were no differences in ctDNA mutation detection rates before start of FOLFIRINOX between patients with disease control (30.0%) and patients with progressive disease (44.4%, *p* = 0.361), or after one cycle of chemotherapy between patients with disease control (16.7%) and patients with progressive disease (27.8%, *p* = 0.468).

The most frequently detected ctDNA mutations were *KRAS* (17/96 samples in total, 12/48 before chemotherapy, 5/48 after 1 cycle of chemotherapy), *TP53* (12/96 samples in total, 8/48 before chemotherapy, 4/48 after 1 cycle of chemotherapy), and *PIK3CA* (4/96 samples in total, 2/48 before chemotherapy, 2/48 after 1 cycle of chemotherapy) mutations. Differences in detection rates between patients with disease control and patients with progressive disease are presented in Table 2. Before start of FOLFIRINOX *TP53* ctDNA mutations were significantly more often detected in patients with progressive disease (33.3%) compared with patients with disease control (6.7%, *p* = 0.040). After chemotherapy no statistically significant differences between disease control and progressive patients were found in detection rates of any of the major tumor mutations. For this reason, only results retrieved from samples collected before FOLFIRINOX will be further discussed.

**Table 2.** Differences in (ctDNA) mutation detection rates between patients with disease control and patients with progressive disease during FOLFIRINOX; *p* values were calculated with Fisher’s exact tests, the value in bold is statistically significant.

	Disease control patients, *n* = 30 (%)	Progressive disease patients, *n* = 18 (%)	*p*	All patients, *n* = 48 (%)
ctDNA mutations detected before the start of FOLFIRINOX				
Any ctDNA mutation	9 (30.0)	8 (44.4)	0.361	17 (35.4)
*KRAS*	6 (20.0)	6 (33.3)	0.325	12 (25.0)
*TP53*	2 (6.7)	6 (33.3)	**0.040**	8 (16.7)
*PIK3CA*	1 (3.3)	1 (5.6)	1.000	2 (4.2)
ctDNA mutations detected after one cycle of FOLFIRINOX				
Any ctDNA mutation	5 (16.7)	5 (27.8)	0.468	10 (20.8)
*KRAS*	2 (6.7)	3 (16.7)	0.349	5 (10.4)
*TP53*	1 (3.3)	3 (16.7)	0.142	4 (8.3)
*PIK3CA*	0 (0)	2 (11.1)	0.136	2 (4.2)

ctDNA, circulating tumor DNA.

There were no differences in detection rates of ctDNA mutations between the different stages of disease (Supplemental Table S2).

### Germline variant detection

Five germline variants were found in multiple patients: *TP53* p.Pro72Arg, *KDR* p.Gln472His, *KIT* p.Met541Leu, *ERBB2* p.Ile625Val, and *PIK3CA* p.Ile391Met. All these germline variants are known single nucleotide polymorphisms (SNPs). In Table 3, the frequencies of all genotypes are presented per response group: disease control or progressive disease. There was no difference in the distribution of the different genotypes (homozygous reference allele, heterozygous, or homozygous mutant allele) between patients with disease control and patients with progressive disease for the germline mutations in *KDR* (*p* = 0.955), *KIT* (*p* = 0.932), *ERRBB2* (*p* = 0.521), and *PIK3CA* (*p* = 0.624). The homozygous *TP53* Pro72Arg variant was more often detected in patients with progressive disease (83.3%) compared with disease control patients (50.0%, *p* = 0.031).

**Table 3.** Differences in genotype frequencies of SNPs in patients with disease control and patients with progressive disease during FOLFIRINOX; *p* values were calculated with Pearson’s Chi-squared tests.

	Disease control patients, *n* = 30 (%)	Progressive disease patients, *n* = 18 (%)	*p*	All patients, *n* = 48 (%)
*TP53* Pro72Arg				
Pro/Pro	2 (6.7)	1 (5.6)	0.056	3 (6.3)
Pro/Arg	13 (43.3)	2 (11.1)		15 (31.3)
Arg/Arg	15 (50.0)	15 (83.3)		30 (62.5)
Pro/Pro + Pro/Arg	15 (50.0)	3 (16.7)	0.031	18 (37.5)
Arg/Arg	15 (50.0)	15 (83.3)		30 (62.5)
*KDR* Gln472His				
Gln/Gln	18 (60.0)	10 (55.6)	0.955	28 (58.3)
Gln/His	9 (30.0)	6 (33.3)		15 (31.3)
His/His	3 (10.0)	2 (11.1)		5 (10.4)
*KIT* Met541Leu				
Met/Met	24 (80.0)	14 (77.8)	0.932	38 (79.2)
Met/Leu	5 (16.7)	3 (16.7)		8 (16.7)
Leu/Leu	1 (3.3)	1 (5.6)		2 (4.2)
*ERBB2* Ile625Val				
Ile/Ile	18 (60.0)	9 (50.0)	0.521	27 (56.3)
Ile/Val	11 (36.7)	7 (38.9)		18 (37.5)
Val/Val	1 (3.3)	2 (11.1)		3 (6.3)
*PIK3CA* Ile391Met				
Ile/Ile	26 (86.7)	17 (94.4)	0.624	43 (89.6)
Ile/Met	3 (10.0)	1 (5.6)		4 (8.3)
Met/Met	1 (3.3)	0 (0)		1 (2.1)

Arg, arginine; Gln, glutamine; His, histidine; Ile, isoleucine; Leu, leucine; Met, methionine; Pro, proline; SNP, single nucleotide polymorphism; Val, valine.

There were no differences in the distribution of germline variant genotypes between the different stages of disease (Supplemental Table S2).

### Predictive value of circulating mutations

Detection of *TP53* ctDNA mutations before start chemotherapy [odds ratio (OR) 7.00, 95% confidence interval (CI) 1.23–39.78, *p* = 0.028] and the presence of a homozygous *TP53* Pro72Arg germline SNP (OR 5.00, 95% CI 1.20–20.92, *p* = 0.028) were predictive factors of progression during FOLFIRINOX (Table 4). There was no collinearity between these factors (VIF 1.0). *TP53* mutations remained significant predictors of tumor progression during FOLFIRINOX after adjusting for stage of disease and baseline CA 19-9 level: OR 10.51 (95% CI 1.40–79.14, *p* = 0.022) for detection of *TP53* ctDNA mutations before start chemotherapy and OR 6.98 (95% CI 1.31–37.30, *p* = 0.023) for a homozygous *TP53* Pro72Arg variant presence.

**Table 4.** Univariable and multivariable binary logistic regression model for the prediction of early tumor progression during FOLFIRINOX; *p* values in bold are statistically significant.

Variable	Univariable		Multivariable	
	OR for tumor progression (95% CI)	*p*	OR for tumor progression (95% CI)	*p*
Stage of disease				
Resectable	Ref			
LAPC	1.20 (0.29–4.91)	0.800		
Metastatic	1.50 (0.36–6.35)	0.582		
CA19-9 at baseline (per 100 kU/l)	1.01 (0.99–1.02)	0.280		
ctDNA mutation detected before start FOLFIRINOX				
No	Ref			
Yes	1.87 (0.55–6.29)	0.314		
*KRAS* ctDNA mutation detected before start FOLFIRINOX				
No	Ref			
Yes	2.00 (0.53–7.54)	0.306		
*TP53* ctDNA mutation detected before start FOLFIRINOX				
No	Ref		Ref	
Yes	7.00 (1.23–39.78)	**0.028**	10.51 (1.40–79.14)	**0.022**
*TP53* Pro72Arg germline variant				
Not homozygous (Pro/Pro or Pro/Arg genotype)	Ref		Ref	
Homozygous (Arg/Arg genotype)	5.00 (1.20–20.92)	**0.028**	6.98 (1.31–37.30)	**0.023**
*KDR* Gln472His germline variant				
No (Gln/Gln genotype)	Ref			
Yes (Gln/His or His/His genotype)	1.20 (0.37–3.91)	0.762		
*KIT* Met541Leu germline variant				
No (Met/Met genotype)	Ref			
Yes (Met/Leu or Leu/Leu genotype)	1.14 (0.27–4.76)	0.854		
*ERBB2* Ile625Val germline variant				
No (Ile/Ile genotype)	Ref			
Yes (Ile/Val or Val/Val genotype)	1.50 (0.46–4.87)	0.500		
*PIK3CA* Ile391Met germline variant				
No (Ile/Ile genotype)	Ref			
Yes (Ile/Met or Met/Met genotype)	0.38 (0.04–3.72)	0.408		

Arg, arginine; CA19-9, carbohydrate antigen 19-9; CI, confidence interval; ctDNA, circulating tumor DNA; Gln, glutamine; His, histidine; Ile, isoleucine; LAPC, locally advanced pancreatic cancer; Leu, leucine; Met, methionine; OR, odds ratio; Pro, proline; Ref, reference value; Val, valine.

Five patients (out of the total cohort of *n* = 48, 10.4%) had both the *TP53* ctDNA mutation before the start of FOLFIRINOX as well as the homozygous Pro72Arg variant present. All five of these patients showed progression during FOLFIRINOX. The combination of both *TP53* mutations detected before the start of FOLFIRINOX showed a sensitivity of 27.8% and specificity of 100% to predict tumor progression during FOLFIRINOX in this cohort. The positive predictive value was 100% and the negative predictive value 69.8%.

### Prognostic value of circulating mutations

Out of 48 patients, 33 (69%) died during follow up. The median follow up of patients alive at last follow up was 16.8 months.

**Figure 1.**
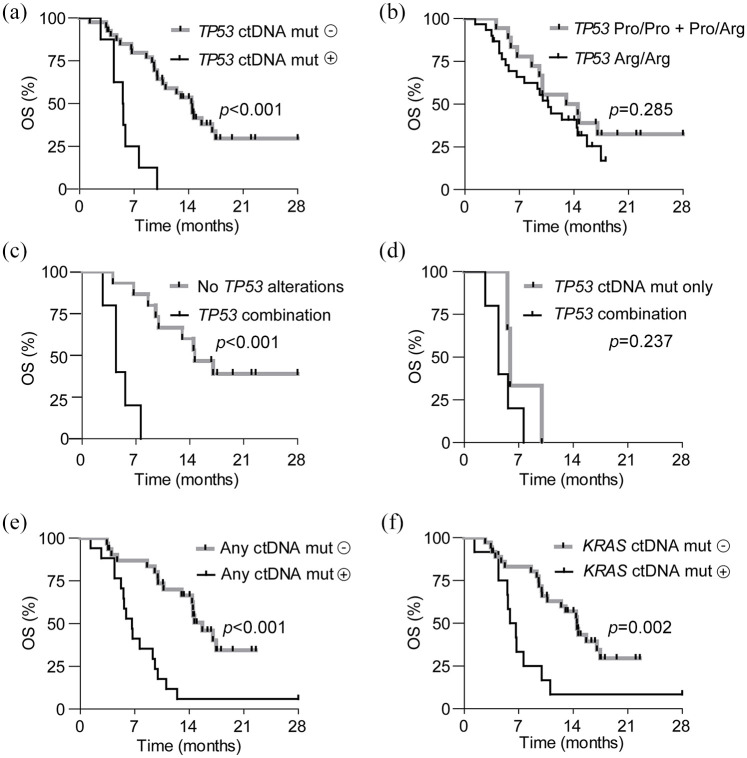
Kaplan–Meier curves for OS for patients with and without circulating mutations detected before the start of FOLFIRINOX; *p* values were calculated with log-rank tests. (a) Patients with (*n* = 8) or without (*n* = 40) a *TP53* ctDNA mutation. (b) Patients with (*n* = 30) and without (*n* = 18) a homozygous germline *TP53* Pro72Arg variant. (c) Patients with the combination of a *TP53* ctDNA mutation and a homozygous germline *TP53* Pro72Arg variant (*n* = 5), and patients without a *TP53* ctDNA mutation and without a homozygous *TP53* Pro72Arg variant (*n* = 15). (d) Patients with the combination of *TP53* mutations (*n* = 5) compared with patients with a *TP53* ctDNA mutation alone (*n* = 3). (e) Patients with (*n* = 17) and without (*n* = 31) any ctDNA mutation. (f) Patients with (*n* = 12) and without (*n* = 32) a *KRAS* ctDNA mutation. Arg, arginine; ctDNA, circulating tumor DNA; Mut, mutation; OS, overall survival; Pro, proline.

Kaplan–Meier curves are shown in Figure 1. Patients with *TP53* ctDNA mutations detected before the start of FOLFIRINOX showed a median OS of 5.6 months (95% CI 3.9–7.2 months). Patients without *TP53* ctDNA mutations had a median OS of 14.5 months (95% CI 11.6–17.3 months, *p* < 0.001), as presented in Figure 1a. Patients with a homozygous *TP53* Pro72Arg variant did not show significantly worse OS (median OS 10.7 months, 95% CI 8.3–13.1 months) compared with patients without this homozygous SNP (median OS 13.0 months, 95% CI 3.6–22.4 months, *p* = 0.285), shown in Figure 1b.

The combination of the presence of a circulating *TP53* ctDNA mutation before the start of FOLFIRINOX with a homozygous *TP53* Pro72Arg germline variant was associated with shorter OS (median OS 4.4 months, 95% CI 2.6–6.2 months) compared with patients without this combination (median OS 13.0 months, 95% CI 8.6–17.4 months, *p* < 0.001) (Figure 1c). Patients with both a *TP53* ctDNA mutation and a homozygous *TP53* Pro72Arg germline variant detected before the start of FOLFIRINOX showed similar OS (median OS 4.4 months; 95% CI 2.6–6.2 months) compared with patients with *TP53* ctDNA mutations alone (median OS 5.9 months; 95% CI 5.4–6.4 months, *p* = 0.237) (Figure 1d). Patients with any ctDNA mutation before the start of FOLFIRINOX, including *TP53* and *KRAS* mutations, did as well show shorter OS (median OS 6.6 months, 95% CI 5.2–8.1 months) compared with patients without any detectable ctDNA mutation (median OS 15.7 months, 95% CI 13.0–18.3 months, *p* < 0.001) (Figure 1e). Patients with a *KRAS* ctDNA mutation detected before the start of FOLFIRINOX showed worse OS (median OS 5.9 months, 95% CI 4.2–7.6 months) than patients without a *KRAS* ctDNA mutation (median OS 14.5 months, 95% CI 12.4–16.5 months, *p* = 0.002) (Figure 1f).

A univariable and multivariable model for OS is presented in Table 5. A *TP53* ctDNA mutation detected before start of FOLFIRINOX was a significant predictor for OS in univariable analysis [hazard ratio (HR) 4.39. 95% CI 1.90–10.13, *p* < 0.001], but not in multivariable analysis.

**Table 5.** Univariable and multivariable Cox proportional hazards model for OS after FOLFIRINOX chemotherapy; *p* values in bold are statistically significant.

Variable	Univariable		Multivariable	
	HR for OS (95% CI)	*p*	HR for OS (95% CI)	*p*
Age (per year)	1.00 (0.96–1.05)	0.932		
Stage of disease				
Resectable	Ref		Ref	
LAPC	1.22 (0.51–2.94)	0.659	1.06 (0.42–2.69)	0.905
Metastatic	2.77 (1.20–6.39)	**0.017**	1.61 (0.65–4.02)	0.308
CA19-9 at baseline (per 100 kU/l)	1.00 (1.00–1.01)	**0.041**	1.00 (1.00–1.00)	0.544
CtDNA mutation detected before start chemotherapy				
No	Ref		Ref	
Yes	4.22 (2.04–8.75)	<**0.001**	4.29 (1.40–13.12)	**0.011**
*KRAS* ctDNA mutation detected before start chemotherapy				
No	Ref		Ref	
Yes	3.16 (1.48–6.71)	**0.003**	0.48 (0.11–1.99)	0.308
*TP53* ctDNA mutation detected before start chemotherapy				
No	Ref		Ref	
Yes	6.26 (2.47–15.87)	<**0.001**	3.30 (0.78–13.92)	0.104
*TP53* Pro72Arg germline variant				
Not homozygous (Pro/Pro or Pro/Arg genotype)	Ref			
Homozygous (Arg/Arg genotype)	1.47 (0.72–3.02)	0.289		
*KDR* Gln472His germline variant				
No (Gln/Gln genotype)	Ref			
Yes (Gln/His or His/His genotype)	0.73 (3.59–1.50)	0.395		
*KIT* Met541Leu germline variant				
No (Met/Met genotype)	Ref			
Yes (Met/Leu or Leu/Leu genotype)	1.81 (0.83–3.92)	0.134		
*ERBB2* Ile625Val germline variant				
No (Ile/Ile genotype)	Ref			
Yes (Ile/Val or Val/Val genotype)	0.96 (0.48–1.91)	0.900		
*PIK3CA* Ile391Met germline variant				
No (Ile/Ile genotype)	Ref			
Yes (Ile/Met or Met/Met genotype)	1.07 (0.38–3.06)	0.893		

Arg, arginine; CA19-9, carbohydrate antigen 19-9; CI, confidence interval; ctDNA, circulating tumor DNA; Gln, glutamine; HR, hazard ratio; His, histidine; Ile, isoleucine; LAPC, locally advanced pancreatic cancer; Leu, leucine; Met, methionine; OS, overall survival; Pro, proline; Ref, reference value; Val, valine.

The presence of any detectable ctDNA mutations before start of FOLFIRINOX remained a significant prognostic factor for OS after adjusting for age, stage of disease, and baseline CA 19-9 level with HR 4.29 (95% CI 1.40–13.12, *p* = 0.011). For the presence of the *TP53* Pro72Arg germline variant, prognostic value could not be demonstrated.

## Discussion

This multicenter pilot study describes the predictive and prognostic value of ctDNA mutations in PDAC patients, detected with next generation sequencing (NGS) before and after one cycle of FOLFIRINOX. We found that circulating *TP53* mutations detected before the start of FOLFIRINOX predict tumor progression during FOLFIRINOX. These mutations include both *TP53* ctDNA mutations and a homozygous *TP53* Pro72Arg germline variant. Furthermore, circulating *TP53* mutations were found to be a poor prognostic factor for OS in PDAC patients treated with FOLFIRINOX. The results of our study suggest that PDAC patients could be spared from ineffective FOLFIRINOX and its side effects by a simple blood draw before the start of treatment.

To our knowledge, *TP53* ctDNA mutations and the common *TP53* Pro72Arg variant have not been described previously for their predictive value for FOLFIRINOX response in PDAC. However, both types of mutations have been described to play a role in cancer development and progression, including in PDAC.^19–21^

It is important to distinguish the prognostic and predictive value of ctDNA mutations from tumor tissue-specific mutations. *KRAS* mutations are present in almost all PDAC tumors, and ~40% of PDAC tumors have *TP53* mutations.^22^ Only a limited number of the mutant PDAC patients, however, have detectable ctDNA mutations: in our cohort, 44%. It is known that PDAC patients with mutant *KRAS* and mutant *TP53* tumors have a worse prognosis compared with patients with wild-type *KRAS* and *TP53* tumors.^23,24^ The prognosis is even worse for patients with detectable ctDNA mutations. The underlying hypothesis for this negative prognostic effect of mutant ctDNA is that aggressive tumors with extensive metastatic properties grow faster, have a high cell death rate, infiltrate (large) blood vessels, and lead to a higher tumor load, thus shed more ctDNA into the circulation.^25^ The prognostic value of detectable ctDNA mutations in patients with PDAC has been described in several studies. The presence of any and/or specific ctDNA mutations, such as *KRAS* and *TP53*, are associated with poor OS and progression-free survival.^26–28^ Our results support this hypothesis: the detection of any ctDNA mutations before the start of FOLFIRINOX was a negative prognostic factor for OS (HR 4.29, 95% CI 1.40–13.12, *p* = 0.011). Moreover, all patients with a detectable *TP53* ctDNA mutation before the start of FOLFIRINOX died from PDAC progression within 10 months. In our cohort, *KRAS* ctDNA mutations were associated with OS in univariable analyses, but not statistically significant in multivariable analyses.

The *TP53* gene is an important tumor suppressor gene. Wild-type *TP53* regulates the cell cycle, initiates apoptosis and senescence, and activates DNA repair in situations of DNA damage and cellular stress, thus inhibiting tumorigenesis.^29^ *TP53* is the second most frequently mutated gene in PDAC and is likely responsible for the susceptibility to cancer development.^22^ In human cancers, missense mutations in *TP53* are the most common type, often leading to gain-of-function and promotion of tumorigenesis.^29,30^ Most somatic *TP53* mutations are located in codons 175, 245, 248, 249, 273, and 282.^30,31^ Others have shown that restoration of wild-type *TP53* in PDAC cell lines with gain-of-function *TP53* mutations enhances the sensitivity to 5-fluorouracil (5-FU), irinotecan, cisplatin, and gemcitabine.^32^ These data support the results of our study: patients without detectable *TP53* ctDNA mutations showed a better response to FOLFIRINOX.

The germline *TP53* Pro72Arg SNP is a well-known variant in the human population. With the replacement of a guanine base by a cytosine base, the accompanying amino acid changes from a proline (Pro) into an arginine (Arg). This amino acid change affects the structure of the protein and might thereby influences its function.^33^ The *TP53* Pro72Arg variant shows varying allele-frequencies in different populations, according to the 1000 Genomes Project.^34^ Because of its high frequency in humans, the SNP *TP53* Pro72Arg has been studied for its association with cancer risk and cancer development in a multitude of studies.^19^ Allele frequencies of this SNP are known to be different in European compared with Asian populations and the combination with ethnicity–specific genetic makeup could lead to different phenotypes. In our Dutch Caucasian PDAC cohort, the Arg/Arg variant is most prevalent: 62.5% of patients show this homozygous variant. Since no healthy controls were included in this experiment, we were not able to assess whether this frequency is different from the healthy Dutch population, and whether the Arg variant is associated with an increased risk of PDAC. For the interpretation of clinical trials, it is very important to keep in mind that genotypes might influence the response to treatment. It cannot be assumed that results are directly applicable to patients from other ethnicities or elsewhere in the world, since allele frequencies of SNPs may differ considerably.

The combination of the *TP53* Pro72Arg variant with a somatic *TP53* mutation in tumor tissues has been described for its poor prognostic value in other cancer types^35^; however not yet in PDAC. It would be relevant to further analyze whether in this population more poor prognostic features could be found with the use of large public databases.

The variety in ctDNA mutation detection methods between different study results makes it difficult to draw general conclusions. For example, Droplet Digital PCR has a higher sensitivity for ctDNA mutation detection than NGS, but can only be used to search for pre-determined specific mutations, such as in *KRAS* codons 12 and 13.^36^ We used a broad 57 gene cancer panel, including more amplicons than only the major PDAC hotspots and covering the entire *TP53* coding region.

For this exploratory study, we included patients with PDAC from all disease stages. We specifically chose this study design for a couple of reasons. First, the distinction between disease stages is often difficult and we believe that the disease stage determined with radiography is a less important factor for the choice of treatment and prognostic outcomes than the molecular biology of PDAC. Second, with the increasing number of clinical trials investigating neoadjuvant (FOLFIRINOX) chemotherapy, including resectable PDAC patients in biomarker studies is required for future personalized treatment. However, we do acknowledge that including patients from different disease stages is also a limitation of the study. Treatment schedules differ between stages of disease. Resectable patients might undergo surgical resection of the tumor, and LAPC patients sometimes receive additional stereotactic body radiation therapy. Although our data does not show differences in detection rates of ctDNA mutations or germline variants between the different disease stages, treatment schedules might have impact on survival differences between or even within disease stages.

The low amount of ccfDNA/ctDNA in PDAC patients compared with patients with other solid cancers, such as lung cancer,^37^ is another important limitation when using broader sequencing techniques. Furthermore, the sensitivity to detect ctDNA mutations using NGS in this study can be improved. It is important, however, to emphasize the stringent method we used to measure ccfDNA concentrations. Instead of the more commonly used, less accurate methods, such as NanoDrop (spectrophotometric) or Qubit (fluorometric), we have used RT-qPCR with Alu115 primers to determine the true ccfDNA concentration, preventing overestimation of ccfDNA quantity.^38^ Moreover, due to the study design of this pilot study, including a relative small number of patients, we did not consecutively select patients for NGS.

This study was designed as a broad, exploratory pilot study, since there is limited literature on possible predictive biomarkers for FOLFIRINOX response in patients with PDAC.^39^ The next step would be to conduct a validation study including a larger patient cohort, focused on *TP53* mutations alone, comparing different treatment regimens to FOLFIRINOX and implementing a more sensitive NGS protocol. By increasing the amount of plasma for DNA isolation, the ccfDNA yield will increase. A higher DNA input amount for sequencing library preparation would increase the probability to detect rare tumor mutations. Last, a molecular barcoding technique with unique molecular identifiers (UMIs) could be used to be able to detect ctDNA mutations at lower allele frequencies without the risk of false positive mutation calling, because errors introduced during library preparation, target enrichment, or sequencing can be filtered out easily.^40^ When including more patients, subgroup analyses on the different tumor stages can be performed in order to check if the predictive value is the same in all PDAC patients receiving FOLFIRINOX. It would be important to see whether treatment response can also be predicted with circulating *TP53* mutations for other types of chemotherapy in PDAC, and if patients that are not responding to FOLFIRINOX could benefit from, for example, gemcitabine-based chemotherapy.

In summary, the combination of a *TP53* ctDNA mutation with a homozygous *TP53* Pro72Arg germline variant is a marker for early tumor progression during FOLFIRINOX and is associated with poor OS. Before translating these results to clinical practice and adjusting treatment decisions, additional cohort studies will be necessary to validate our findings.

## Supplemental Material

sj-docx-1-tam-10.1177_17588359211033704 – Supplemental material for Circulating TP53 mutations are associated with early tumor progression and poor survival in pancreatic cancer patients treated with FOLFIRINOX Click here for additional data file. Supplemental material, sj-docx-1-tam-10.1177_17588359211033704 for Circulating TP53 mutations are associated with early tumor progression and poor survival in pancreatic cancer patients treated with FOLFIRINOX by Fleur van der Sijde, Zakia Azmani, Marc G. Besselink, Bert A. Bonsing, Jan Willem B. de Groot, Bas Groot Koerkamp, Brigitte C. M. Haberkorn, Marjolein Y. V. Homs, Wilfred F. J. van IJcken, Quisette P. Janssen, Martijn P. Lolkema, Saskia A. C. Luelmo, Leonie J. M. Mekenkamp, Dana A. M. Mustafa, Ron H. N. van Schaik, Johanna W. Wilmink, Eveline E. Vietsch and Casper H. J. van Eijck in Therapeutic Advances in Medical Oncology
