# Supplementary material for: Circulating TP53 mutations are associated with early tumor progression and poor survival in pancreatic cancer patients treated with FOLFIRINOX
Source: Ther Adv Med Oncol. 2021 Aug 18;13:17588359211033704. doi: 10.1177/17588359211033704 (PMC8377319; doi:10.1177/17588359211033704)
Supplement: sj-docx-1-tam-10.1177_17588359211033704 – Supplemental material for Circulating TP53 mutations are associated with early tumor progression and poor survival in pancreatic cancer patients treated with FOLFIRINOX [file sj-docx-1-tam-10.1177_17588359211033704.docx]

**Supplementary Table 1.** Genes included in Accel-Amplicon 57G Plus Pan-Cancer Profiling Panel

| *ABL1* | *EZH2* | *JAK3* | *PTPN11* |
| --- | --- | --- | --- |
| *AKT1* | *FBXW7* | *KDR* | *RB1* |
| *ALK* | *FGFR1* | *KIT* | *RET* |
| *APC* | *FGFR2* | *KRAS* | *SMAD4* |
| *ATM* | *FGFR3* | *MAP2K1* | *SMARCB1* |
| *BRAF* | *FLT3* | *MET* | *SMO* |
| *CDH1* | *FOXL2* | *MLH1* | *SRC* |
| *CDKN2A* | *GNA11* | *MPL* | *STK11* |
| *CSF1R* | *GNAQ* | *MSH6* | *TP53** |
| *CTNNB1* | *GNAS* | *NOTCH1* | *TSC1* |
| *DDR2* | *HNF1A* | *NPM1* | *TSC2* |
| *DNMT3A* | *HRAS* | *NRAS* | *VHL* |
| *EGFR* | *IDH1* | *PDGFRA* |  |
| *ERBB2* | *IDH2* | *PIK3CA* |  |
| *ERBB4* | *JAK2* | *PTEN* |  |

*Full exon coverage

**Supplementary Table 2.** Differences in circulating tumor DNA (ctDNA) mutation and germline variant detection rates between patients with different disease stages. *P-*values were calculated with Pearson’s Chi-squared tests.

|  | **Resectable disease patients n=18 (%)** | **Locally advanced patients n=16 (%)** | **Metastatic disease patients n=14 (%)** | ***P*** |
| --- | --- | --- | --- | --- |
| **ctDNA mutations detected before the start of FOLFIRINOX** | | | | |
| Any ctDNA mutation | 4 (22.2) | 5 (31.3) | 8 (57.1) | 0.112 |
| *KRAS* | 3 (16.7) | 3 (18.8) | 6 (42.9) | 0.184 |
| *TP53* | 2 (11.1) | 2 (12.5) | 4 (28.6) | 0.363 |
| *PIK3CA* | 2 (11.1) | 0 (0) | 0 (0) | 0.176 |
| **ctDNA mutations detected after one cycle of FOLFIRINOX** | | | | |
| Any ctDNA mutation | 3 (16.7) | 6 (37.5) | 1 (7.1) | 0.107 |
| *KRAS* | 2 (11.1) | 3 (18.8) | 0 (0) | 0.243 |
| *TP53* | 2 (11.1) | 2 (12.5) | 0 (0) | 0.403 |
| *PIK3CA* | 2 (11.1) | 0 (0) | 0 (0) | 0.176 |
| **Germline variants** | | | | |
| *TP53* Pro72Arg  Pro/Pro  Pro/Arg  Arg/Arg  Pro/Pro + Pro/Arg  Arg/Arg | 1 (5.6  5 (27.8)  12 (66.7)  6 (33.3)  12 (66.7) | 2 (12.5)  4 (25.0)  10 (62.5)  6 (37.5)  10 (62.5) | 0 (0)  6 (42.9)  8 (57.1)  6 (42.9)  8 (57.1) | 0.579  0.859 |
| *KDR* Gln472His  Gln/Gln  Gln/His  His/His | 10 (55.6)  6 (33.3)  2 (11.1) | 9 (56.3)  5 (31.3)  2 (12.5) | 9 (64.3)  4 (28.6)  1 (7.1) | 0.983 |
| *KIT* Met541Leu  Met/Met  Met/Leu  Leu/Leu | 14 (77.8)  3 (16.7)  1 (5.6) | 13 (81.3)  3 (18.8)  0 (0) | 11 (78.6)  2 (14.3)  1 (7.1) | 0.886 |
| *ERBB2* Ile625Val  Ile/Ile  Ile/Val  Val/Val | 10 (55.6)  6 (33.3)  2 (11.1) | 8 (50.0)  7 (43.8)  1 (6.3) | 9 (64.3)  5 (35.7)  0 (0) | 0.719 |
| *PIK3CA* Ile391Met  Ile/Ile  Ile/Met  Met/Met | 18 (100)  0 (0)  0 (0) | 14 (87.5)  1 (6.3)  1 (6.3) | 11 (78.6)  3 (21.4)  0 (0) | 0.142 |

Arg = arginine, Gln = glutamine, His = histidine, Ile = isoleucine, Leu = leucine, Met = methionine, Pro = proline, Val = valine.


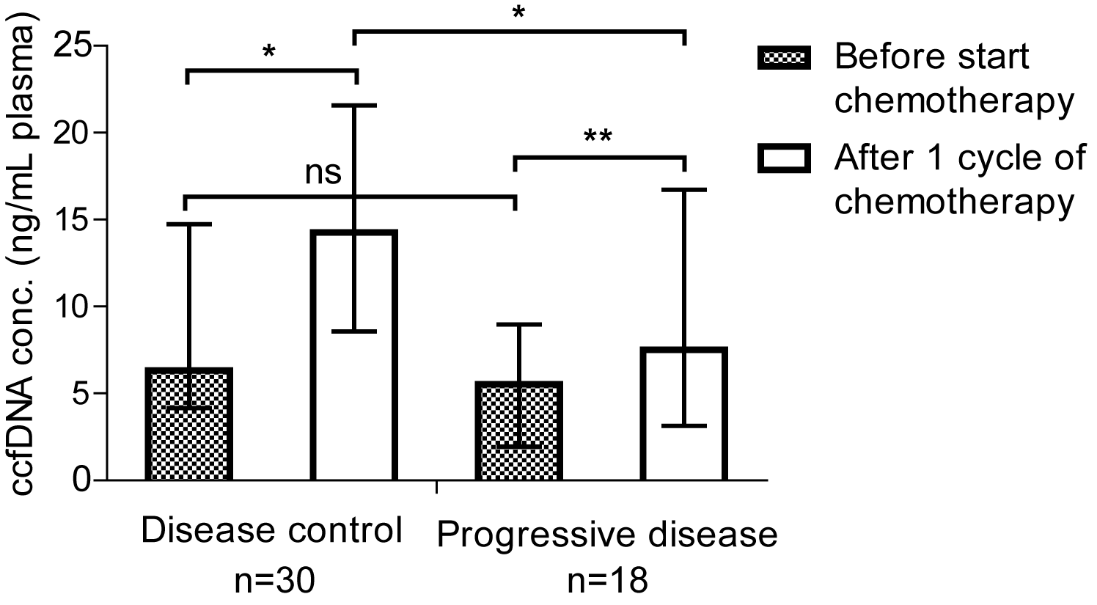


**Supplementary Figure 1.** Circulating cell-free DNA (ccfDNA) concentration dynamics in pancreatic cancer patients during one cycle of FOLFIRINOX.

Concentrations (conc.) of ccfDNA were corrected for short fragment sized DNA, based on Alu115 primer RT-qPCR measurements. Data are presented as medians with interquartile ranges. Wilcoxon signed rank tests were used to compare ccfDNA concentrations before and after chemotherapy, Mann-Whitney U tests to compare DNA concentrations between disease control and progressive disease patients (ns = not significant, * = *P*<0.05, ** = *P*<0.01).

**
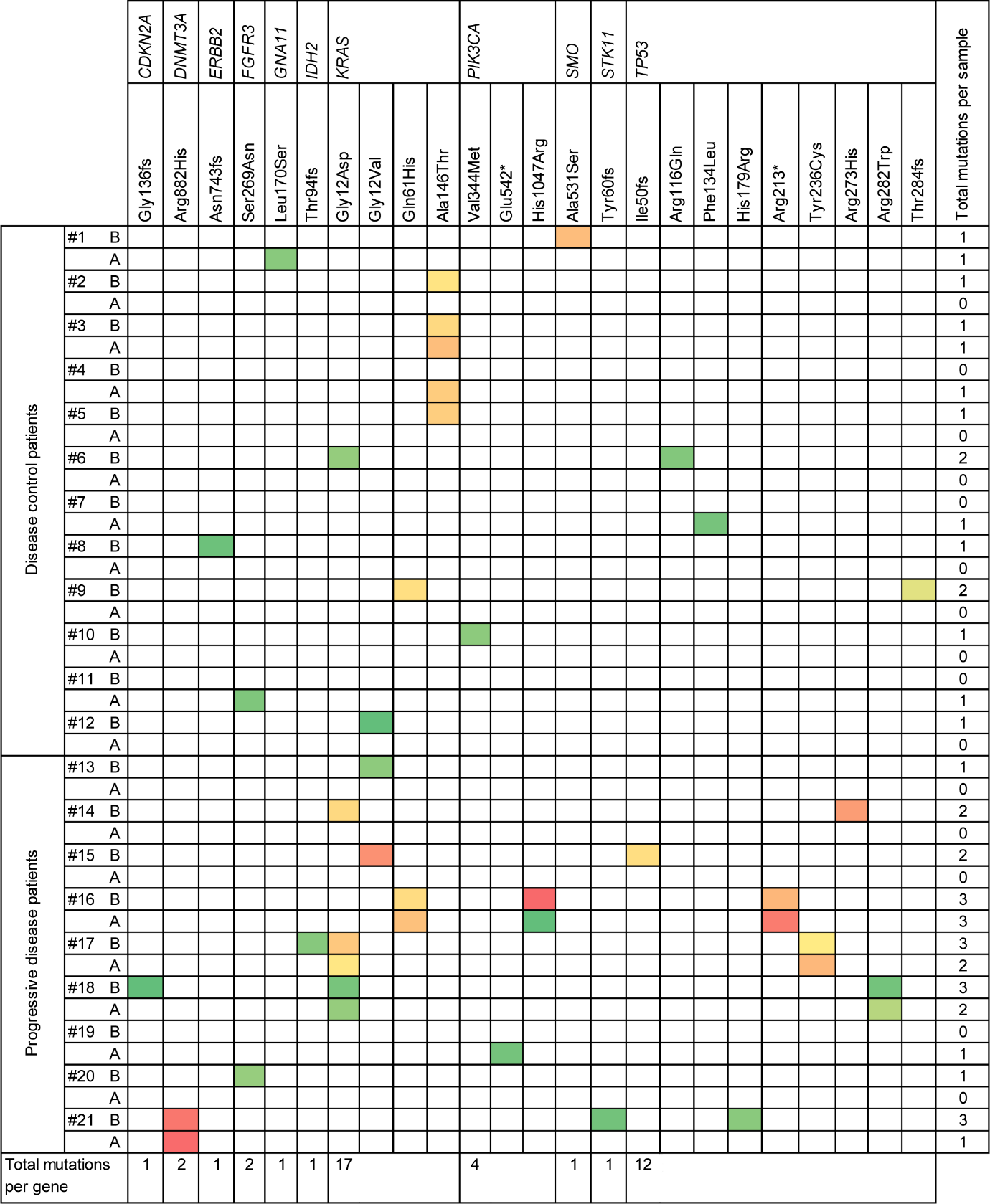
**

**Supplementary Figure 2.** Variant allele frequencies (VAF) of circulating tumor DNA (ctDNA) mutations detected per patient sample.

21/48 patients had at least one detectable ctDNA mutation in one of the samples (before or after one cycle of chemotherapy).

B = before start of FOLFIRINOX, A = after one cycle of FOLFIRINOX, fs = frame shift, * = stop codon. Color legend: green is lowest, yellow is median, red is highest VAF (range: 1.02-7.56%).
